# A gender-specific geodatabase of five cancer types with the highest frequency of occurrence in Iran

**DOI:** 10.1186/s13104-024-06737-4

**Published:** 2024-03-19

**Authors:** Sharareh Faramarzi, Behzad Kiani, Mohammedreza Hoseinkhani, Neda Firouraghi

**Affiliations:** 1 Department of Medical Informatics, School of Medicine, Mashhad University of Medical Sciences, Mashhad, Iran; 2 UQ Centre for Clinical Research, Faculty of Medicine, The University of Queensland, Brisbane, Queensland Australia

**Keywords:** Cancer incidence, Cancer epidemiology, Spatial epidemiology, Public health, Geographic Information Systems (GIS), Iran

## Abstract

**Objectives:**

Cancer is a global health challenge with complex characteristics. Despite progress in research and treatment, a universally effective prevention strategy is lacking. Access to reliable information, especially on occurrence rates, is vital for cancer management. This study aims to create a database containing individual and spatially integrated data on commonly diagnosed cancers in Iran from 2014 to 2017, serving as a valuable resource for spatial-epidemiological approaches.

**Data description:**

This database encompasses several files related to cancer data. The first file is an Excel spreadsheet, containing information on newly diagnosed cancer cases from 2014 to 2017. It provides demographic details and specific characteristics of 482,229 cancer patients. We categorized this data according to the International Agency for Research on Cancer (IARC) reporting rules to identify cancers with the highest incidence. To create a geodatabase, individual data was integrated at the county level and combined with population data. Files 2 and 3 contain gender-specific spatial data for the top cancer types and non-melanoma skin cancer. Each file includes county identifications, the number of cancer cases for each cancer type per year, and gender-specific population information. Lastly, there is a user’s guide file to help navigate through the data files.

## Objective

Cancer has emerged as a notable focal point within public health across various communities, standing as the third primary contributor to mortality [1]. Regrettably, there has been a surge in cancer occurrences in recent times, amplifying its importance as a foremost concern within the healthcare domain [2]. The impact of cancer extends beyond developed nations and encompasses low- and middle-income countries as well, where resources for prevention, early detection, and treatment are often inadequate. Roughly 70% of global cancer cases are concentrated in these lower- and middle-income nations [3, 4]. Annually, over 50,000 new cases are identified within the Iranian population [5]. The living conditions of individuals can have a significant impact on their overall health. Cancer trends can vary among different populations and regions, highlighting the influence of factors such as work-related and industrial settings [6], socioeconomic situation [7], healthcare accessibility [8], and environmental exposures that play a crucial role on cancer incidence [9, 10]. This wide variety of factors influencing cancer incidence showcases the dynamic, multidisciplinary nature of cancer research.

The geographical variation and the impact of environmental factors underscore the importance of spatial analysis in understanding patterns across both space and time. Spatial analysis of diseases contributes to recognize high-risk areas and patterns of occurrence to provide evidence-based information in order to enact efficient screening and disease management strategies in areas with high priority [11–16]. Utilizing spatial techniques such as local Moran *I*, hot spot analysis, and spatiotemporal scanning can aid in identifying high-risk zones. This approach can help generate hypotheses for further analysis on the relationships between risk factors and cancer incidence, providing valuable insights into the connections between environmental hazards, individual lifestyles, and cancer incidence within communities [17, 18].

In this research, we utilized Geographic Information System (GIS) to create a geodatabase encompassing high prevalent cancer cases in Iran between 2014 and 2017. This database is a set of individual and spatial data which can serve as a valuable resource for identifying spatial and temporal patterns of high-risk and low-risk areas for cancer incidence. In forthcoming studies, the geodatabase can be combined with socio-environmental factors such as poverty rate, income and lifestyle factors to assess the risk factors [13]. By integrating these datasets, researchers can take proactive measures in resource allocation and tailor interventions to specific geographic locations with higher cancer risks. This geodatabase might be useful for both spatial epidemiological research and machine learning algorithms in terms of classification or clustering studies.

## Data description

This data was collected across the entire country of Iran, located in western Asia. Cancer has become the third most prominent cause of death in the country, with increasing in occurrence in recent years. This rise can be attributed to the country’s rapid advancements in industrialization and modernization, as well as significant changes in people’s lifestyles and environment. These transformations have the potential to impact the occurrence and distribution of different types of cancer [19–21]. Incidence rates of cancer may vary across different geographic locations, potentially due to differing environmental factors [22]. Lifestyle [23] and environmental factors play significant roles in contributing to this phenomenon [24]. Pesticides and industrial chemicals have been linked to an increased risk of cancer [25, 26]. Urbanization causes air pollution, and sedentary lifestyles, and increased exposure to carcinogens [27, 28]. Climate variations and geographic characteristics can affect cancer rates, with regions experiencing increased ultraviolet radiation exposure having higher rates of skin cancer [29]. Moreover, lifestyle factors such as smoking, diet, physical activity, and alcohol consumption can all influence cancer risk and may vary by geographic location [30, 31] and areas with lower socioeconomic status may have higher rates of cancer due to factors such as limited access to healthcare, and unhealthy living conditions [28, 32].

In this study the data was obtained for entire country from three different sources. Population data was gathered from Iran’s statistical center, through the most recent national census in 2016 [33]. According to this data, Iran had an estimated total population of 80 million. The country boundaries, in the county scale, were provided by the Ministry of the Interior in vector map shapefile format. Iran is composed of 417 counties and 31 provinces, encompassing a total land area of 1,648,195 km [33]. During the years 2014–2017, 482,229 cancer cases data were obtained from the Iranian National Population-Based Cancer Registry (INPCR) [34]. The INPCR records newly diagnosed cases of cancer with malignant primary tumors. In the case of metastatic cancers, the focus is on tracing back to the primary tumor, and only information about the primary tumor is recorded for that patient. Tumor topography, morphology, and grade are coded in this registry using the third edition of the International Classification of Diseases for Oncology (ICD-O) [35].

Population-based cancer data has been collected from various sources including death certificates, clinical investigations such as X-ray, endoscopy, imaging, ultrasound, exploratory surgery (such as laparotomy), cytology, and pathology. To ensure accuracy and completeness, strategies like training staff, following standardized procedures and guidelines (based on ICD-O, the International Agency for Research on Cancer (IARC), and WHO guidelines), conducting audits, utilizing validation checks, and comparing data with other sources are implemented [21]. The university cancer registry secretariat uses the Sima-ye-Saratan system to process and control the quality of data. They check for duplicate records and ensure internal consistency before submitting the data to the national registry. Patient information is entered and checked for duplicates before tumor information is added [34].

This database includes 3 data files and a help file (Table 1). Data file 1 includes individual data of cancer cases diagnosed between 2014 and 2017, over the whole country. This data contains sex, age, diagnosis year, code of tumor topography, code of tumor morphology and behavior, code of tumor grade, source of diagnosis report, and county ID. We conducted an examination to prepare the gender-specific spatial data of top cancer types in the matter of incidence. The individual data has been carefully categorized according to IARC reporting rules, and we have identified the five cancer types with the highest incidence rates in Iran, in addition to non-melanoma skin cancer. It is noteworthy that non-melanoma skin cancer is often excluded from global cancer case counts. The exclusion is primarily due to its widespread occurrence and predominant treatment within primary healthcare facilities, contributing to potential under-reporting in national cancer registry data [36]. Furthermore, we have geocoded and linked this data to the county level boundaries as a geographical reference and incorporated population data as well.

Data file 2 includes spatial data for five cancer types with the highest frequency of occurrence and non-melanoma skin cancer in females. The cancer types covered are breast, non-melanoma skin cancer, thyroid, stomach, colon and brain and nervous system. This file provides aggregated data based on county boundaries and includes county identification number and name, geographical coordinates (longitude and latitude), number of cancer cases for each cancer per year, total and females’ population. Data file 3 is assigned for spatial data of top cancer types in males. These files include stomach, non-melanoma skin cancer, prostate, bladder, trachea, bronchial, lung cancer (TBL) and colon cancer. Similar to the previous file, each file comprises aggregated data on cancer cases and includes county identification number and name, geographical coordinates, number of cancer type per year, total and males’ population. These data files are in shapefile format which is a digital format used for storing both the geometric location and relevant attribute information of vector-based data. It is commonly used for spatial data storage [37]. Data file 4 is a help file in Microsoft Excel format that provides a description of the fields used in the previous files. This file includes two sheets, first one has been designed to assist in understanding and utilizing the data from other files and the second sheet contains the list of counties ID and names. The data does not include any identification data of patients.

These data files can be used by researchers in different disciplines such as spatial epidemiology, health and cancer epidemiology, public health, and health service research. These data can be used for spatial visualization (hotspot analysis [38, 39], and local Moran’s clustering [40], spatio-temporal analysis such as purely temporal, purely spatial, spatial-temporal, and spatial variation to detect spatial patterns of different types of cancer [14, 41]. By incorporating sociodemographic and environmental variables, a regression model or artificial intelligence methods can be employed to explore and understand the correlation between cancer incidence rates and lifestyle or environmental factors. GIS is well-equipped to integrate diverse data from multiple sources, including spatial, temporal, and descriptive elements, within a unified framework. Table 1 presents the specifics of each dataset and offers access links to these data.

**Table 1:** Dataset overview

Label	Name of data file	File types (File extension)	Data repository and identifier
Data file 1	Individual data of cancer cases	MS Excel file (*.xlsx)	Harvard Dataverse, (10.7910/DVN/7ZK41X) [42]
Data file 2	Spatial data of top cancer types in females	Polygonal features shapefile format (*.shp)	Harvard Dataverse, (10.7910/DVN/7ZK41X) [43]
Data file 3	Spatial data of top cancer types in males	Polygonal features shapefile format (*.shp)	Harvard Dataverse, (10.7910/DVN/7ZK41X) [44]
Data file 4	Help file	MS Excel file (*.xlsx)	Harvard Dataverse, (10.7910/DVN/7ZK41X) [45]

## Limitation

The data recorded and reported by INPCR had a delay of 4 years, which means the most recent data is not available to us. Additionally, the prevalence of non-melanoma skin cancer, often treated in primary healthcare settings, may lead to under-reporting in national cancer registry data. Another limitation we encountered was the lack of an annual census in Iran. Instead, we had to rely on the latest census conducted in 2016.

## Data Availability

The data described in this data note can be freely and openly accessed on Harvard Data-verse (10.7910/DVN/7ZK41X) [46]; consisting of four datafiles: Data file 1 (10.7910/DVN/7ZK41X)[42], Data file 2 (10.7910/DVN/7ZK41X)[43], Data file 3 (10.7910/DVN/7ZK41X)[44], Data file 4 (10.7910/DVN/7ZK41X)[45]. Additional information can be found in Table 1.

## Abbreviations

GIS: Geographic Information Systems
INPCR: Iranian National Population-Based Cancer Registry
ICD-O: International Classification of Diseases for Oncology
IARC: International Agency for Research on Cancer
TBL: Trachea, Bronchus, Lung
